# Integrated metabolomic and transcriptomic analyses reveal that glutamine promotes chicken primordial germ cell-like cell specification potentially through α-ketoglutarate

**DOI:** 10.5713/ab.260260

**Published:** 2026-07-27

**Authors:** Qingqing Geng, Lei Zhang, Zhe Wang, Kunyu Liang, Fufu Cheng, Jing Chen, Yifan Xi, Xinyue Jiao, Junqiang Hou, Farooq Mazhar, Sheng Cui, Yani Zhang

**Affiliations:** 1 College of Veterinary Medicine, Yangzhou University, Yangzhou, China; 2 Key Laboratory of Animal Breeding Reproduction and Molecular Design for Jiangsu Province, College of Animal Science and Technology, Yangzhou University, Yangzhou, China; 3 College of Animal Science and Technology, Jiangsu Agri-Animal Husbandry Vocational College, Taizhou, China

**Keywords:** α-ketoglutarate, Amino Acids, Glutamine Metabolism, Primordial Germ Cell, Primordial Germ Cell-like Cell

## Abstract

**Objective:**

Primordial germ cells (PGCs) are essential for germline transmission and preservation of genetic resources in poultry. Despite identification of several genes and signaling pathways involved in PGC specification, the upstream regulatory mechanisms, including metabolic regulation and epigenetic modification, remain poorly understood. This study aimed to characterize the metabolic landscape underlying chicken PGC development and to identify key metabolic regulators involved in BMP4-induced specification of primordial germ cell-like cells (PGCLCs) *in vitro*.

**Methods:**

Untargeted metabolomics and transcriptomics were performed to examine metabolic profiles and gene expression patterns between chicken embryonic stem cells (ESCs) and PGCs. Differential metabolites and metabolism-related genes were identified through integrated multi-omics analysis. Functional experiments, including glutamine deprivation and metabolite supplementation, were conducted to verify the role of glutamine metabolism in PGCLC specification.

**Results:**

A total of 194 differentially expressed metabolites were identified, mainly enriched in amino acid, nucleotide, energy metabolism, and antioxidant pathways. Compared with ESC, PGC contained elevated levels of amino acids, peptides, and benzoic acid derivatives, while carbohydrates were more abundant in ESC. Transcriptomic analysis of 14,405 annotated genes revealed similar metabolic gene expression patterns in male and female samples. Integrated analysis indicated that amino acid metabolism, particularly glutamine metabolism, is crucial in PGC formation. Glutamine deprivation significantly inhibited PGCLC specification, disrupted the balance between glycolysis and oxidative phosphorylation, and altered H3K4me2 epigenetic modification. Supplementation with the glutamine-derived tricarboxylic acid cycle metabolite α-ketoglutarate (α-KG) markedly restored the reduced efficiency of PGCLC specification caused by glutamine depletion.

**Conclusion:**

Glutamine promotes the specification of chicken PGCLC and correlates with alterations in cellular metabolism and increased H3K4me2 levels during differentiation. These findings suggest a potential association between glutamine metabolism, α-KG, and epigenetic regulation in germ cell development, providing new insights into the metabolic basis of PGC formation and a foundation for improving PGC culture and expansion in poultry.

## INTRODUCTION

Primordial germ cell (PGC) in chickens are precursor cells for the formation of sperm and oocytes during development, and their proper development directly determines the maintenance of reproductive capacity [1,2]. The formation of PGC is regulated by multiple factors in the embryonic microenvironment, including signaling pathways, epigenetic modifications, and metabolic states. To date, researchers have identified key signaling pathways, such as Wnt/β-Catenin and TGF-β/BMP, which play significant roles in coordinating the proliferation, specialization, and migration of PGC [3–5]. Key transcriptional regulators, such as bone morphogenetic protein-4 (BMP4) and basic fibroblast growth factor (bFGF), have been shown to play important roles in shaping the proliferation dynamics of PGC [5–7]. The determination of PGC fate also involves epigenetic reprogramming, such as the demethylation of 5-methylcytosine (5-mC), histone H3 lysine 4 trimethylation (H3K4me3), and Histone H3 lysine 4 dimethylation (H3K4me2) [8–10]. Interestingly, changes in epigenetic patterns, including modifications of histones and DNA, are believed to be intricately linked to metabolic regulation [11]. In particular, α-ketoglutarate (α-KG), a key intermediate of the tricarboxylic acid (TCA) cycle, has emerged as an important metabolic–epigenetic regulator that links cellular metabolism to chromatin remodeling and cell fate determination through its role as a cofactor for α-KG-dependent dioxygenases [12]. However, despite existing research on molecular signaling and transcriptional regulation in PGC development, its metabolic regulatory mechanisms remain underexplored.

Metabolism is a critical regulatory factor in germ cell development. Nutritional conditions in the culture medium and the pregnant maternal environment, along with metabolic pathways, influence the formation and development of fetal germ cells [13–15]. These conditions not only affect the basic energy supply required by the cells but also have profound effects on the proliferation, migration, and final differentiation of germ cells by regulating key metabolic pathways. In mice, inhibition of fatty acid oxidation impairs the homing of spermatogonial stem cells, preventing their correct localization to the basal membrane of the seminiferous tubules and causing apoptosis of mislocalized spermatogonial stem cells at P7 [16]. Additionally, methylenetetrahydrofolate reductase, an enzyme in the Serine-Glycine-One-Carbon Metabolism Pathway, when deficient, results in impaired sperm development [17]. Moreover, betaine, as a methyl donor in methionine metabolism, significantly affects spermatogenesis, sperm count, and fertility in mice [18]. Therefore, optimizing culture medium composition and ensuring good maternal nutritional status are not only crucial for fetal reproductive health but may also provide potential strategies for improving reproductive-related diseases. While some studies have shown that certain metabolites are related to PGC development, research on the metabolic landscape during PGC formation is still very limited, particularly in terms of the diversity of metabolites and their regulatory mechanisms.

The combination of metabolomics and transcriptomics provides new tools to study metabolic regulation in germ cell development [19]. These technologies comprehensively reveal dynamic changes in metabolites and their pathways during cell development while analyzing gene expression changes. Previous studies have conducted integrated metabolomic and proteomic analyses of PGC [20], revealing that the major energy metabolic pathway during PGC formation shifts from glycolysis to oxidative phosphorylation (OXPHOS). Additionally, another study used untargeted metabolomics to compare the metabolic features of epiblast-like cell (EpiLC) and primordial germ cell-like cell (PGCLC). The results showed an increased ratio of pyruvate to lactate in PGCLC, with elevated levels of TCA cycle intermediates and glutamate [21]. These findings further highlight the central role of metabolism in determining germ cell fate. However, current research on metabolic remodeling during PGC development has primarily focused on mammals, with limited studies in chickens.

Therefore, understanding the influence of key metabolites on PGC formation is an important entry point for understanding the connection between nutrition and health. Based on this background, this study integrates untargeted metabolomics and transcriptomics technologies to systematically compare the differential metabolites and associated metabolic pathways between embryonic stem cell (ESC) and PGC. We focused on amino acid metabolism changes during PGCLC formation and explored the potential roles of key differentially expressed metabolites (DEMs), such as glutamine and its derivatives, in influencing PGCLC specification. These findings not only contribute to elucidating the metabolic mechanisms of reproductive health but also provide scientific evidence for clinical nutritional interventions to improve reproductive capacity.

## MATERIALS AND METHODS

### Cell isolation and culture

This experiment utilized the Rugao yellow chicken as the animal model, provided by the Poultry Research Institute of Jiangsu Province. The incubation conditions were set at a temperature of 37°C and humidity of 60%. Stage X blastoderms were collected for ESC isolation and purification, and genital ridges obtained on 5.5 d for PGC isolation, as detailed in earlier studies [22].

Chicken fibroblasts (DF-1) cells were cultured in Dulbecco’s modified Eagle medium (DMEM, 21013024; Gibco) with high glycemic index, supplemented with 10% fetal bovine serum (FBS, 16140071; Gibco), 100 U/mL penicillin, and 100 U/mL streptomycin (15140148; Gibco). For ESC and PGC, knockout (KO)-DMEM (A3181501; Gibco) was employed, including 10% FBS, 2.5% chicken serum (C5405; Sigma-Aldrich), 100 U/mL penicillin, 100 U/mL streptolysin, 0.4 μmol/L non-essential amino acids (NEAAs; M7145; Sigma-Aldrich), 2 mmol/L glutamine (G7513; Sigma-Aldrich), 0.1 mmol/L β-mercaptoethanol (M6250; Sigma-Aldrich), 10 ng/μL mouse leukemia inhibitory factor (mLIF, ESG1106; Sigma-Aldrich), 10 ng/μL bFGF (GF446; Sigma-Aldrich), and 5 ng/mL human stem cell factor (hSCF, GF021; Sigma-Aldrich). ESC differentiation into PGC was induced *in vitro* using high-glucose DMEM without glutamine (11960044; Gibco) as the basal medium, supplemented with BMP4 protein. The complete composition of the differentiation medium for each experimental condition is detailed in Table 1 [23].

### Assay detects glutamine concentration

To assess the concentration of glutamine in ESC and PGC, we employed a glutamine assay kit (mlbio, ml965581V). Samples were centrifuged at 1,000×g for 20 min, and particulates were promptly removed before proceeding with the assay. Introduction of 50 μL of Standard or Sample to the appropriate wells followed, with the addition of 100 μL of Enzyme conjugate to standard and sample wells (excluding the blank well). The wells were covered with an adhesive strip and incubated for 60 min at 37°C. Four washes were performed on the Microtiter Plate, using a squirt bottle to fill each well with Wash Solution (1×) and aspirating contents into a sink, repeating this process four times. After the final wash, the plate was inverted, tapped onto absorbent paper or towels until dry, and the washer was optimized to aspirate maximum liquid, setting the fill volume at 350 μL/well/wash. The plate was inverted again and blotted dry. Subsequently, 50 μL of Substrate A and 50 μL of Substrate B were added to each well, gently mixed, and incubated for 15 min at 37°C, shielded from light. The introduction of 50 μL of Stop Solution into each well led to a color change from blue to yellow. If the well color appeared green or the change was uneven, a gentle tap on the plate ensured thorough mixing. The optical density (OD) was read at 450 nm within 15 min using a microtiter plate reader. The x-axis featured the standard substance’s OD value, while the y-axis displayed its concentration. This data was plotted on graph paper or software, allowing for the creation of a standard curve and the derivation of a linear regression equation. The sample’s OD value was then input into the equation to determine its concentration.

### Assay detects glutamic acid concentration

The concentration of glutamic acid in ESC and PGC was assessed using a glutamine assay kit (BC1580; Solarbio). Begin by collecting cells, centrifuging, and discarding the supernatant. Introduce 1 mL of reagent 1 per 10 million cells and disrupt them using ultrasonic waves (power 200 W, ultrasonic for 3 s, 10 s interval, repeat 30 times). Subsequently, centrifuge at 7,000×g at room temperature for 10 min, then take the supernatant for measurement. Dilute the standards into solutions of 0.5, 0.4, 0.3, 0.2, 0.1, 0.05, and 0.025 μmol/mL, respectively. In a 1mL quartz cuvette, mix 200 μL of the standard solution, 800 μL of reagent three, and 50 μL of reagent four. Immediately record the absorbance values A_1_ at 340 nm for 20 s and A_2_ at 5 min and 20 s. Calculate ΔA Standard = A_2_–A_1_. In another 1 mL quartz cuvette, mix 200 μL of the sample, 800 μL of reagent three, and 50 μL of reagent four. Immediately record the absorbance values A_1_ at 340 nm for 20 s and A_2_ at 5 min and 20 s. Calculate ΔA = A_2_–A_1_. Use glutamic acid content (μmol/mL) as the x-axis and ΔA standard as the y-axis to draw a standard curve y = kx+b. Incorporate ΔA from the measurement tube into the equation, where glutamic acid content (μmol/10^4^ cell) = x×V sample÷(1,000÷total V sample×V sample) = 0.001x. Here, V sample total represents the volume of the added extraction solution (1 mL), V sample is the added sample volume (0.2 mL), Cpr is the sample protein concentration (mg/mL), W is the sample mass (g), and 1,000 is the total cell count (10 million). Calculate the x value (μmol/mL) using the equation.

### Measurement of α-ketoglutarate dehydrogenase activity

The α-ketoglutarate dehydrogenase (α-KGDH) activity was assessed utilizing the α-KGDH Assay Kit (BC0715; Solarbio). Initially, 0.8 mL of ddH_2_O was added to reagent 8, ensuring complete dissolution before use. Subsequently, reagents 4, 5, 6 and 7 were mixed with reagent 3 for thorough mixing and dissolution. Next, 0.1 g of fresh sample was placed in-to a centrifuge tube, followed by the addition of 1 mL of reagent 1 and 10 μL of reagent 3, and thorough shaking. The homogenate underwent centrifugation, and the resulting supernatant was transferred to a new centrifuge tube. After centrifuging (11,000×g) for an additional 10 min at 4°C, the supernatant was quantified to determine α-KGDH activity.

Preheating ELIASA BC0715 (Solarbio) for 30 min and adjusting the wave-length to 340 nm were the initial steps. In 96-well plates, 200 μL of reagent 3 (combined with reagents 4, 5, 6, and 7) was dispensed and incubated at 25°C for 10 min. Subsequently, 8 μL of reagent 8 and 12 μL of the previously extracted supernatant solution were added and thoroughly mixed. The absorbance values A_1_ at 0 min and A_2_ at 2 min were then determined, with △A = A_2_−A_1_. A blank control, ensuring test accuracy, followed the same procedure, substituting the supernatant with ddH_2_O. The absorbance values a_1_ at 0 min and a_2_ at 2 min were determined, with △a = a_2_−a_1_. The calculation formula for α-KGDH activity (U/g) was expressed as ([△A−△a]/[ɛ×d]×V_1_×109)/(V_2_/V_3_×W)/T = 22,480.7×(△A−△a)/W, where V_1_ represents the total reaction volume; ɛ signifies the extinction coefficient per mole of NADH; d is the optical path of the 96-well plates; V_2_ denotes the total volume of the extracted supernatant solution (12 μL); V_3_ represents the total volume of reagent 1 and reagent 2 (1.01 mL); W indicates the sample weight (0.1 g); and T is the reaction time (2 min).

### Pyruvate kinase activity measurements

The pyruvate kinase (PK) activity was assessed utilizing the PK Assay Kit (BC0545; Solarbio). Follow the guidelines outlined in the PK activity detection kit manual. Harvest 5 million cells each of ESC and PGC, introduce 1 mL of extraction solution, and disrupt using ultrasonic waves (utilize an ice bath, set power to 20%, apply ultrasonication for 3 s with a 10 s interval, repeating the process 30 times). Subsequently, centrifuge the samples at 4,450×g for 10 min at 4°C, retrieve the supernatant, and maintain it on ice for testing. Add 10 samples, 10 μL of reagent 3, and 180 μL of reagent 2 to the respective wells of a 96-well plate. After thorough mixing, record the absorbance value A_1_ at 340 nm and the absorbance value A_2_ after 3 min. Calculate PK activity using the formula: PK = 5.36 (A_2_–A_1_).

### Broadly targeted metabolomics analysis

#### Sample preparation and extraction

Prior to isolating ESC, a thorough washing and disinfection process was applied to freshly fertilized eggs. Stage X blastoderms were collected to isolate and purify ESC, while genital ridges were obtained on 5.5 d for PGC isolation. Sexing was performed on individual ESCs during isolation via polymerase chain reaction (PCR), following a previously described protocol [24]. Briefly, the sex-chromosomal CHD1 gene (Z/W) was amplified using Mighty Amp DNA polymerase, and PCR products were visualized by agarose gel electrophoresis. Primers were designed based on the chicken CHD1-Z (chrZ: 51359549-51400046) and CHD1-W (chrW: 4989932-5105612, galGal6a, UCSC) genomic sequences. The expected amplicon sizes were 580 bp for CHD1-Z (chrZ: 51387236-51387815) and 434 bp for CHD1-W (chrW: 5019696-5020129). Primer sequences were: forward, 5′-CTGCGAGAACGTGGCAACAGAGT-3′; reverse, 5′-ATTGAAATGATCCAGTGCTTG-3′. For extensive targeted metabolomic analysis, three samples from each group (ESC male, ESC female, PGC male, PGC female) were equally mixed, resulting in 100 individuals per sample. These mixed samples served as quality control (QC) samples, with one QC sample inserted every six formal samples. The samples underwent a series of steps, including transfer to glass vials with pre-chilled methanol-water (V:V = 4:1), addition of chloroform, ice bath sonication, and supplementation with an internal standard (L-2-chlorophenyl alanine). The resulting supernatant, obtained after sonication and centrifugation, was evaporated to dryness in an LC-MS injection vial. The dried sample was reconstituted with methanol-water (V:V = 1:4), stored at −20°C for 2 h, and centrifuged. The resulting supernatant was used for subsequent analysis.

#### Ultra-high performance liquid chromatography-electrospray ionization tandem mass spectrometry conditions and data analysis

The analyzed samples underwent ultra-high performance liquid chromatography-electrospray ionization tandem mass spectrometry (UPLC-ESI-QTRAP-MS/MS) analysis, utilizing the Waters Acquity UPLC HSS T3 C18 column at 40°C, a flow rate of 0.4 mL/min, and an injection volume of 2 μL. The solvent system used was water (0.1% formic acid): acetonitrile (0.1% formic acid), with a gradient program of 95:5 v/v at 0 min, 10:90 v/v at 11.0 min, 10:90 v/v at 12.0 min, 95:5 v/v at 12.0 min, and 95:5 v/v at 14.0 min. The QTRAP mass spectrometer was controlled by Analyst 1.6.3 software, and the ESI source operating parameters were optimized. Metabolite identification utilized public databases, and a specific set of multiple reaction monitoring (MRM) transitions was monitored based on elution periods. The resulting metabolite mass peaks were integrated by peak area, and the analysis of metabolite mass peaks in different samples was conducted. QC samples were prepared by mixing sample extracts to monitor the repeatability of samples analyzed under the same treatment. FastQC (https://www.bioinformatics.babraham.ac.uk/projects/fastqc/) was employed to assess the quality of the original and trimmed sequencing libraries.

### Statistical and bioinformatic analysis

Utilizing the Metware Cloud Platform (https://cloud.metware.cn/), a comprehensive multivariate statistical analysis was conducted, encompassing Wayne analysis, un-supervised principal component analysis (PCA), hierarchical cluster analysis (HCA), and supervised multiple regression orthogonal partial least squares discriminant analysis (OPLS-DA). Differential metabolites between groups were identified through OPLS-DA. The variable importance in projection (VIP) values derived from the OPLS-DA model were used to evaluate the contribution of each metabolite to group separation. Metabolites with a VIP value≥1, an absolute |log_2_(FoldChange)|≥1, and a p<0.05 were considered significantly differential metabolites. The identified differential metabolites underwent annotation using the Kyoto Encyclopedia of Genes and Genomes (KEGG) database (http://www.kegg.jp/kegg/compound/). Pearson’s correlation analysis was employed to examine the relationships between distinct metabolites in ESC and PGC.

### RNA extraction, complementary DNA library preparation, and RNA sequencing

According to the manufacturer’s instructions, total RNA was isolated from ESC male, PGC male, ESC female, and PGC female samples using the Total RNA Isolation Kit (Tiangen). RNA samples for each group were obtained from three biological replicates, with each replicate consisting of 100 individuals. The samples were initially homogenized, followed by the addition of RNA extraction reagent, and processed according to the steps outlined in the kit’s manual. RNA purity and concentration were measured using a NanoDrop 2000 spectrophotometer (Thermo Fisher Scientific). To further assess the integrity of the RNA samples, QC was performed using an Agilent 2100 Bioanalyzer (Agilent Technologies). All libraries were constructed by Biotech and underwent rigorous quality checks. After the sequencing library construction, high-throughput sequencing with pairedend 150 bp reads was performed using the Illumina NovaSeq 6000 platform (Illumina).

To ensure high-quality sequencing data for subsequent analysis, the raw data obtained from the Illumina sequencing platform was preprocessed. FastQC (https://www.bioinformatics.babraham.ac.uk/projects/fastqc/) was used to evaluate the quality of the raw data, analyzing read length distribution, GC content, sequence duplication levels, and adapter contamination, and removing low-quality sequences. Cutadapt (ver. 1.15) was then used to trim adapter sequences and low-quality reads. The processed data were further analyzed using FastQC to perform QC, ensuring that Q20 and Q30 scores met the standards, and that GC content was within the expected range to avoid any impact on subsequent analyses. Next, HISAT2 (ver. 2.0.5) was used to align the clean data to the reference genome GRCg6a (RefSeq accession: GCF_ 000002315.5) downloaded from NCBI, for subsequent expression and differential analysis. Finally, the data was normalized using the fragments per kilobase per million fragments (FPKM) method to ensure comparability of expression levels across different samples and eliminate the impact of technical variations on the analysis results.

### Differential gene expression analysis

Differential gene expression analysis was performed using the DESeq2 package (R language, ver. 1.24.0). First, gene expression data for each group of samples was normalized using DESeq2, and a negative binomial distribution model was applied for differential gene expression analysis. Genes with a p<0.05 and |log_2_(FoldChange)|≥1 were selected as differentially expressed genes (DEGs) for further analysis. To ensure data quality, PCA was performed on the sequencing data, and boxplots were generated to assess the distribution and detect outliers. A volcano plot was then used to visualize the number and distribution of significantly DEGs between ESC and PGC samples from both male and female groups. In this study, up-regulated DEGs were defined as genes significantly higher expressed in ESC relative to PGC, and down-regulated DEGs referred to genes with elevated expression in PGC compared with ESC, consistent with the coloring standard of volcano plot.

Gene Ontology (GO) enrichment analysis was then performed to classify the DEGs functionally in ESC and PGC samples. To further explore DEGs in the amino acid metabolic pathways across comparison groups, a Venn diagram was created to display the shared and unique DEGs. The STRING database (https://www.string-db.org/) was then used to conduct a protein-protein interaction (PPI) network analysis of the overlapping DEGs related to the amino acid metabolic pathway, revealing their potential interactions. Finally, GO enrichment analysis of the overlapping DEGs was conducted to annotate their biological functions and involved pathways, providing a basis for further biological interpretation.

### Integrated analysis of differential metabolites and differentially expressed genes

MetaboAnalyst (https://www.metaboanalyst.ca/) was used to integrate the analysis of DEGs and DEMs to construct a network of metabolic pathways. Relevant metabolic pathways were identified, and the relationships between the DEGs and metabolites within these pathways were analyzed. Visualization tools were employed to display the association network between key metabolites and genes, revealing their potential interactions within specific metabolic pathways.

### Cell counting kit-8 and 5-ethynyl-2′-deoxyuridine staining test

To evaluate the effect of glutamine or α-KG on cell viability, DF-1 cells were plated in 96-well microplates at a density of 5,000 cells per well. After treatment, each well received cell counting kit-8 (CCK-8) solution (10 μL CCK-8/100 μL medium) and was incubated at 37°C for 2 h. Microplate reader absorbance measurements were taken at 450 nm with a reference wavelength. Cell proliferation was assessed using an 5-ethynyl-2′-deoxyuridine (EdU) kit (C10310-1; RiboBio). DF-1 cells, at a density of 2×10^5^ cells per well, were cultured in 6-well plates following the manufacturer’s instructions for the assays. The experiment was performed three times, and statistical comparisons were made using the ratio of the experimental group to the control group.

### Quantitative real-time polymerase chain reaction

Cells were collected at 2, 4 and 6 d following BMP4 induction in the model, representing different treatment conditions. On 5.5 d of *in vivo* incubation, genital ridge samples were obtained. RNA extraction, utilizing the TRIzol method, was followed by reverse transcription to obtain complementary DNA (cDNA) for quantitative real-time polymerase chain reaction (qRT-PCR). The expression of pertinent genes (*CVH*, *C-KIT*, *BLIMP1*, *IDH* and *LDH*) during PGCLC specification was assessed through qRT–PCR, following the instructions of the SuperReal color fluorescent quantitative premix reagent (SYBR Green, fp215-02; Tiangen). mRNA levels were normalized against β-actin, and relative quantification was performed using the 2^−ΔΔCt^ method as described by Livak and Schmittgen [25], conducted in Microsoft Excel. The primer details are available in Table 2.

### Immunofluorescence

On 4 d of the BMP4 induction model, cells treated differently underwent two washes with phosphate-buffered saline (PBS). After washing, cells were fixed with 200 μL of 4% paraformaldehyde for 15 min, followed by two PBS washes. Subsequently, cells were exposed to 200 μL of 0.5% Triton X-100 (vT8200; Solarbio) for 20 min at room temperature. Then, a blocking step was performed using 10% FBS-PBS (10099141; Gibco) for 30 min at room temperature. Primary antibodies, anti-DDX4/MVH (1:300; ab13840; Abcam), were diluted and applied to cover the cell surface at 4°C overnight. PBST was used to remove the primary antibody, and subsequent steps included the addition of goat anti-rabbit IgG (1:1,000; a22120; Abbkine; fluorescein isothiocyanate [FITC] labeled). Incubation was carried out in a cassette for 2 h, followed by washing with PBST. DAPI (C1002; Beyotime) in 200 μL was added, incubated for 15 min, and then washed. Fluorescence observation and photography were conducted using a fluorescence inverted microscope (FV1200; Olympus).

### Flow cytometry

Cells induced by BMP4 for 4 d were collected and centrifuged at 300×g for 6 min. The resulting cell pellet was then resuspended in DDX4/MVH (1:300; ab13840; Abcam) antibody dilution and incubated at 37°C for 2 h. Afterward, the cells underwent centrifugation at 300×g for 6 min, followed by three washes in PBST. Incubation with goat anti-rabbit IgG (1:1,000; a22120; Abbkine; FITC labeled) was carried out at 37°C for 2 h in the dark to facilitate the detection of CVH-positive cells by flow cytometry (FC).

Cell populations, induced for 4 d with varying glutamine concentrations, were harvested and subjected to lysis using RIPA buffer (R0010; Solarbio) for protein extraction. Following protein concentration determination, 20 μg of total cellular protein was combined with 5 μL of sample buffer and boiled for 3 to 5 min to induce protein denaturation. Subsequently, proteins were separated using a 12% Sure PAGETM precast gel (E304-01; Vazyme), transferred to nitrocellulose membranes, semi-dried, and blocked with 5% skimmed milk at room temperature for 2 h. Membranes were then incubated overnight at 4°C with primary antibodies against H3K4me2 and H3 (ab32356; Abcam). After PBST washing, Goat Anti-Rabbit IgG HampL (HRP; Abcam) was applied and incubated at 37°C for 2 h. Finally, the membranes were imaged and photographed using a BIO-RAD imager (733BR2897).

### Data analysis

All experiments were conducted with three independent biological replicates, each defined as an independently prepared batch of cells from separate differentiations or subcultures. Student’s t-test was used for two-group comparisons, and one-way ANOVA followed by Tukey’s HSD post-hoc test for multi-group analyses. For metabolomic and transcriptomic analyses, raw p-values were reported for initial feature selection (p<0.05 and |log_2_(FoldChange)|≥1). All statistical analyses were performed using SPSS 26.0.

## RESULTS

### Identification of differentially expressed metabolites between embryonic stem cell and primordial germ cell

**Figure 1:**
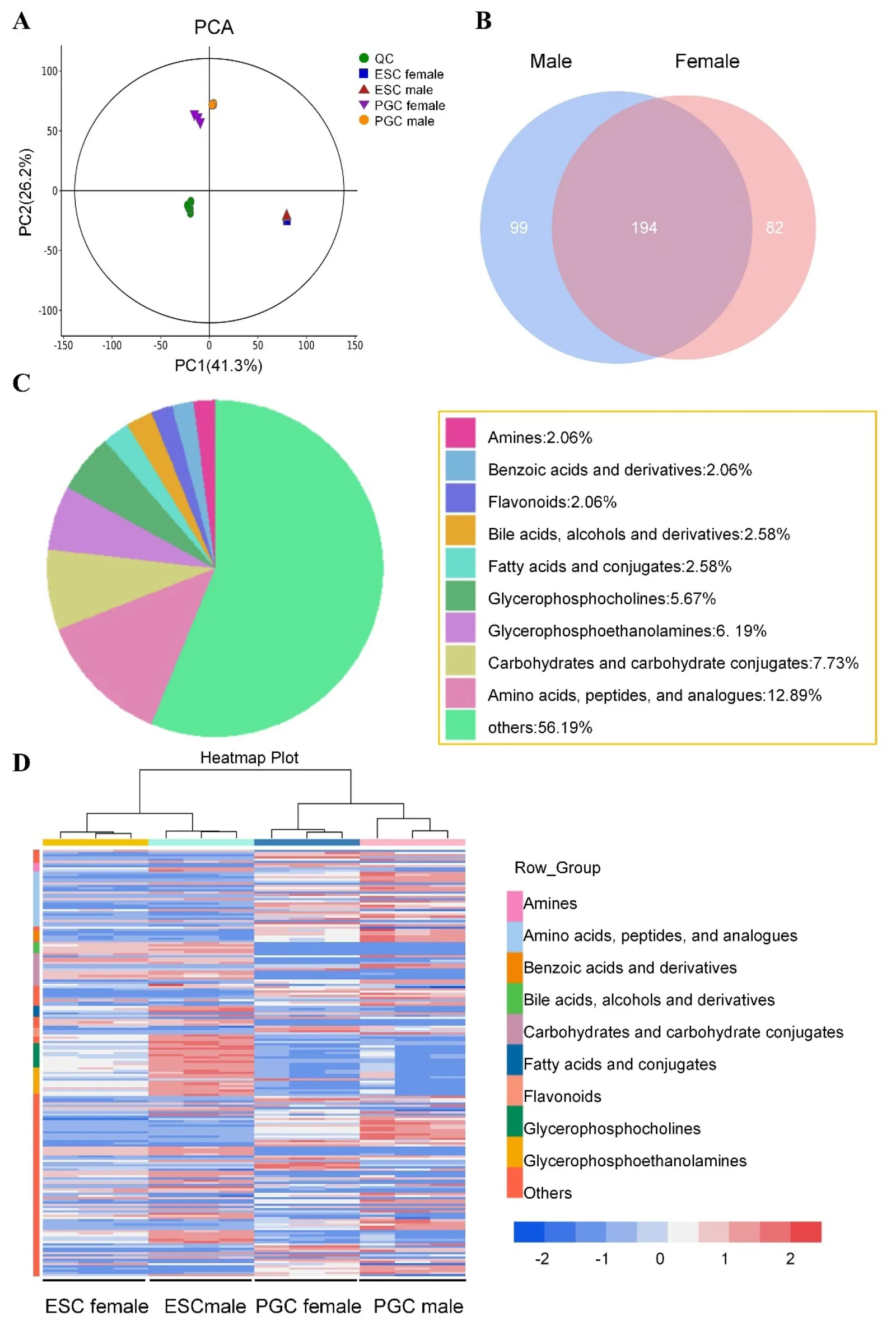
Comprehensive overview of differential metabolites during the differentiation of ESC into PGC. (A) Unsupervised principal component analysis (PCA) of ESC and PGC with three biological replicates. (B) Wayne analysis of differential metabolites in male and female samples. (C) Categorization of 194 metabolites into 12 distinct sample categories. (D) Heatmap illustrating the expression patterns of metabolites in ESC and PGC. QC, quality control; ESC, embryonic stem cell; PGC, primordial germ cell.

A comprehensive identification of metabolites in ESC and PGC was conducted using widely targeted ultra-high performance liquid chromatography-tandem mass spectrometry (UPLC-MS/MS) metabolomics analysis. After data integration (including retention time, exact mass, and MS/MS fragment information), MS1 and MS2 data were compared with known compounds in public databases (such as METLIN, NIST, Mass Bank, KEGG, and m/z Cloud) as well as in a self-built library to achieve precise metabolite identification. The metabolite identification and abundance data are provided in Supplement 1. PCA results showed that the QC group samples were tightly clustered, with the total PC score being 67.5% (PC1 = 41.3%, PC2 = 26.2%) (Figure 1A). The high dispersion between groups indicated changes in metabolites, while the clustering of samples within each group confirmed the good reproducibility and high stability of the method. This suggests a clear distinction in metabolic characteristics between ESC and PGC.

Based on the screening criteria of p<0.05 and |log2 (FoldChange)|≥1, differential metabolites between ESC and PGC were analyzed. Venn analysis was performed to exclude gender-related factors influencing metabolic characteristics, identifying and annotating 194 differential metabolites (Figure 1B). Among the 194 identified DEMs compounds, they were categorized into ten classes based on a threshold of more than three replicates, including 4 amines, 25 amino acids, peptides, and analogues, 4 benzyl alcohols and derivatives, 5 bile acids, alcohols and derivatives, 15 carbohydrates and their conjugates, 5 fatty acids and their conjugates, 4 flavonoids, 11 glycerophosphocholines, and 12 glycerophosphoethanolamines (Figure 1C). A clustering heatmap further demonstrated the differences in metabolite expression between ESC and PGC (Figure 1D). Specifically, compared to ESC, PGC exhibited significantly higher levels of amino acids, peptides, and analogues, as well as benzoic acids and derivatives. On the other hand, carbohydrates and carbohydrate conjugates were found to be significantly higher in ESC than in PGC.

### Significant changes in amino acid metabolism between embryonic stem cell and primordial germ cell

**Figure 2:**
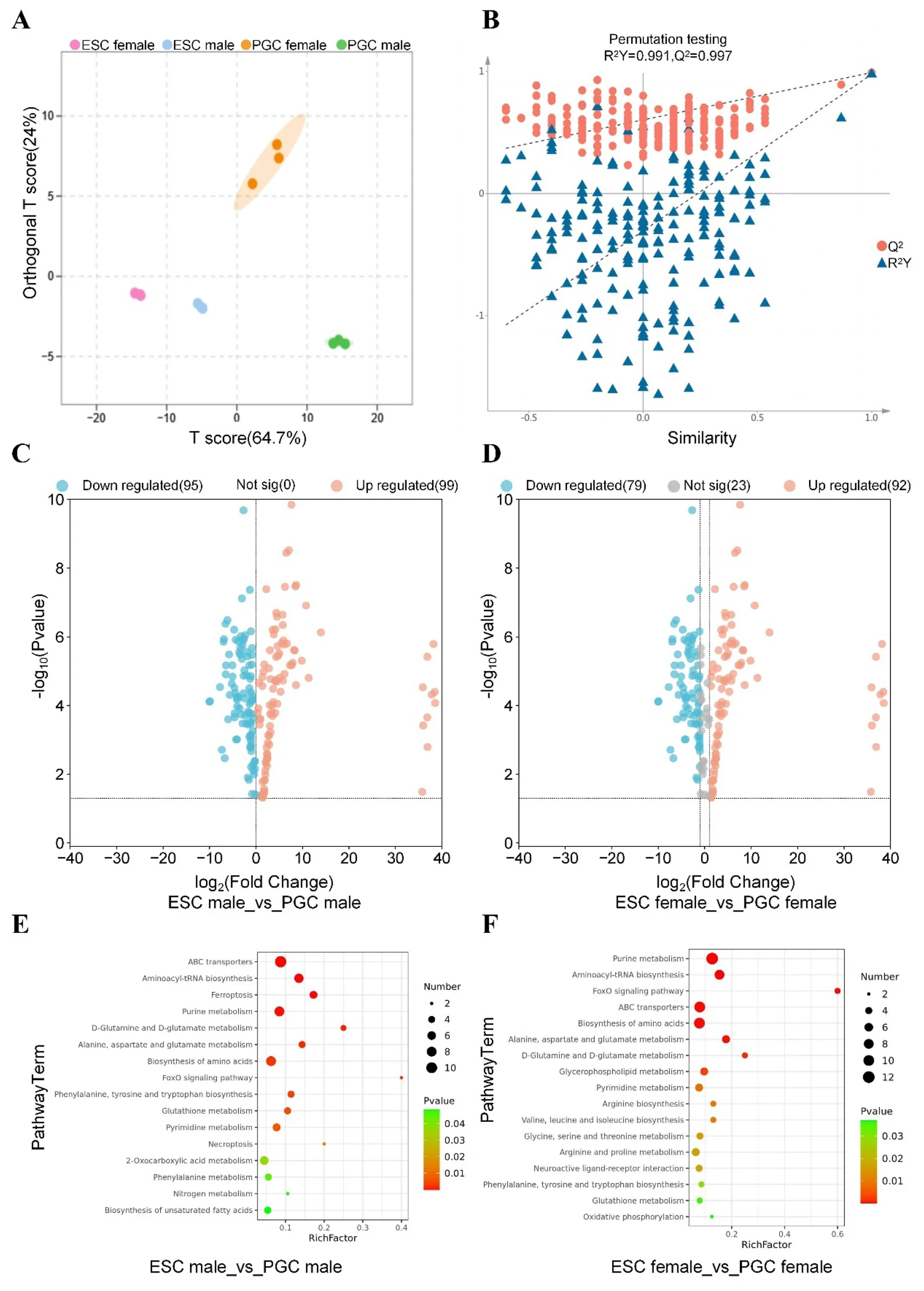
Screening of the key differential metabolite during the formation of PGC. (A) OPLS-DA model showing the key DEMs between ESC and PGC. (B) OPLS-DA score plot showing distinct separation between ESC and PGC samples. Volcano plot showing the differential metabolites between ESC male_vs_PGC male (C) and ESC female_vs_PGC female (D) comparisons. KEGG pathway enrichment analysis of shared differential metabolites between ESC male_ vs_ PGC male (E) and ESC female_ vs_ PGC female (F) comparisons. ESC, embryonic stem cell; PGC, primordial germ cell; OPLS-DA, orthogonal partial least squares discriminant analysis; DEMs, differentially expressed metabolites; KEGG, Kyoto Encyclopedia of Genes and Genomes.

Next, OPLS-DA was performed to identify key DEMs between ESC and PGC (Figure 2A). In the OPLS-DA score plot, ESC and PGC samples showed clear separation (Figure 2A), and the R^2^Y and Q^2^ scores of the model were 1 and 0.994, respectively, indicating a high degree of fit and predictive ability (Figure 2B). Volcano plot analysis revealed that the common differential metabolites in male and female samples exhibited similar expression patterns (Figures 2C, 2D). Meanwhile, KEGG analysis of these differential metabolites showed that the top three enriched pathways shared by male and female samples were the FoxO signaling pathway and Glutamine and Glutamate metabolism (Figures 2E, 2F).

**Figure 3:**
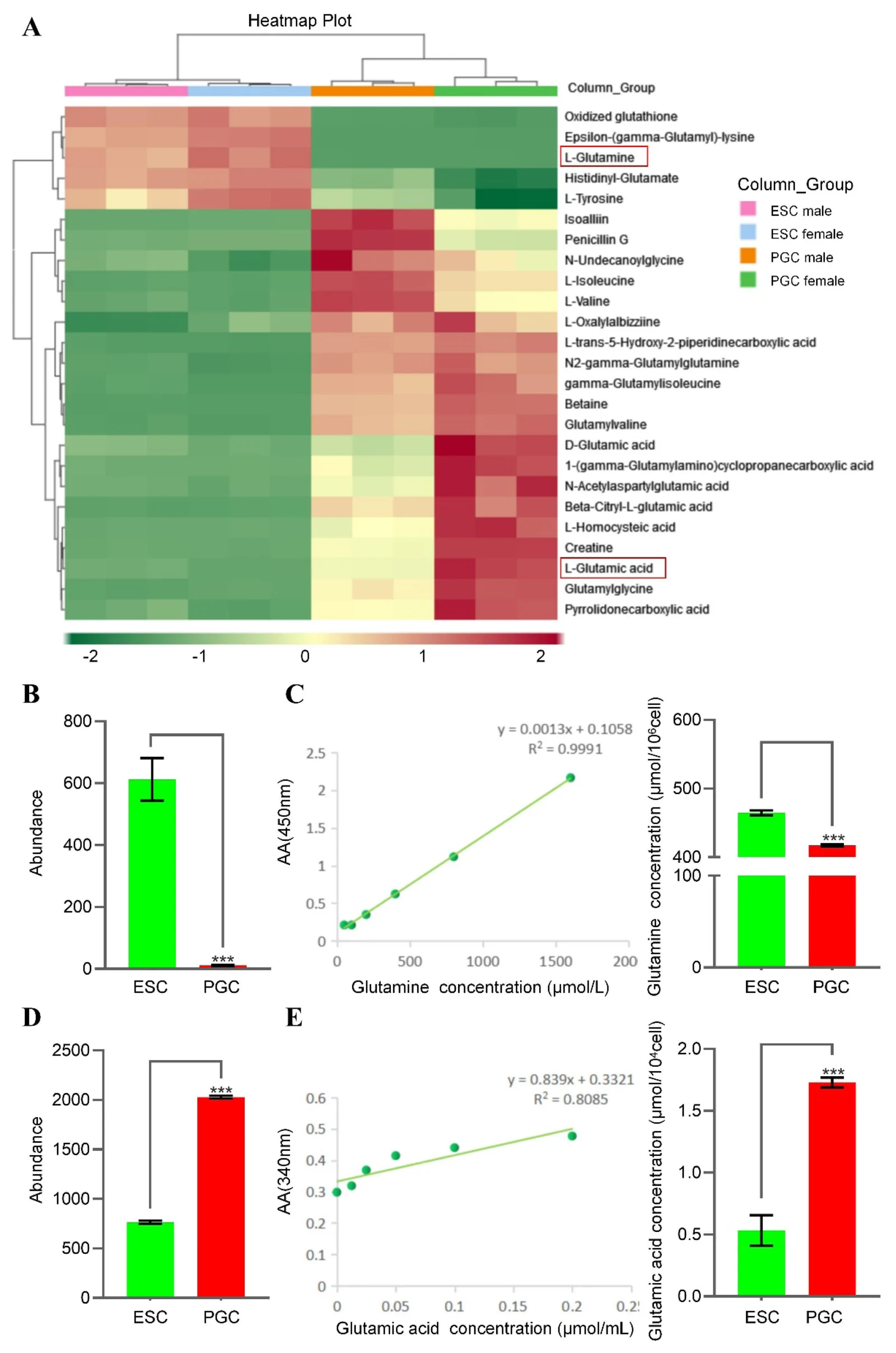
Glutamine and glutamate are differentially expressed in ESC and PGC. (A) Heatmap of hierarchical clustering analysis of amino acid metabolites based on UPLC-MS/MS analysis. (B) Comparison of glutamine expression levels between ESC and PGC. (C) ELISA validation of glutamine expression levels in ESC and PGC. (D) Comparison of glutamate expression levels between ESC and PGC. (E) ELISA validation of glutamate expression levels in ESC and PGC. Data are shown as mean±SEM, n = 3 independent experiments; *** p<0.001 by Student’s t-test. ESC, embryonic stem cell; PGC, primordial germ cell; UPLC-MS/MS, ultra-high performance liquid chromatography-tandem mass spectrometry; SEM, standard error of the mean.

Subsequently, the study selected amino acid-related metabolites based on the condition |log2(FoldChange)|≥2. The relative content of each amino acid metabolite was calculated using the average peak response area via UPLC-MS/MS, and a heatmap for hierarchical clustering analysis was generated (Figure 3A). Interestingly, the metabolomics results showed that, compared to ESC, the expression of glutamine was significantly reduced in PGC (Figure 3B; *** p<0.001); in contrast, the expression of glutamic acid, a downstream metabolite of glutamine, was significantly higher in PGC than in ESC (Figure 3D; *** p<0.001). Furthermore, the ELISA results were consistent with the sequencing results (Figures 3C, 3E; *** p<0.001). This suggests that glutamine metabolism may play a significant biological role in the differentiation of ESC into PGC.

### Differentially expressed genes in embryonic stem cell and primordial germ cell

**Figure 4:**
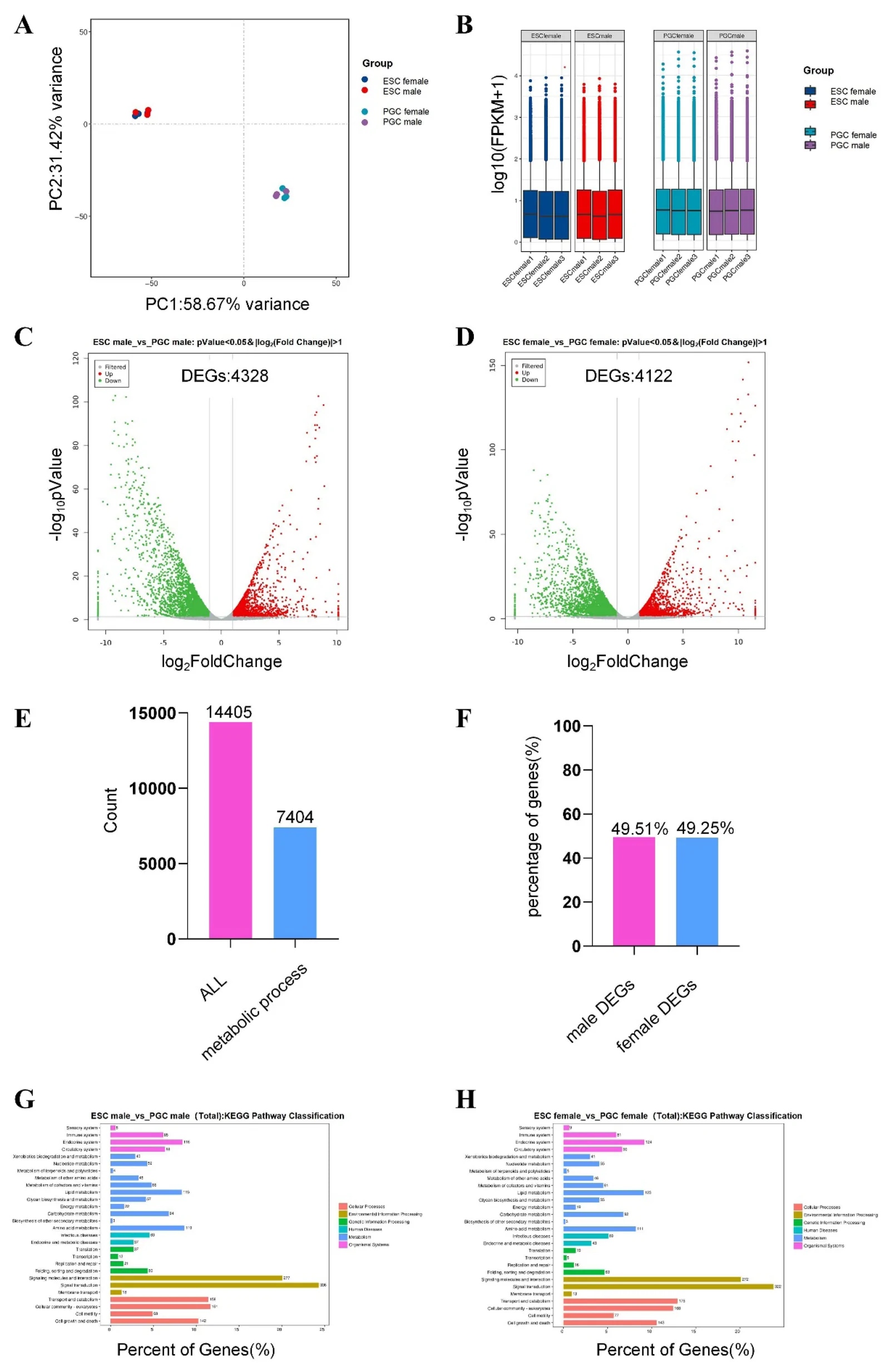
Transcriptomic analysis of ESC and PGC. (A) Principal component analysis (PCA) of RNA sequencing data from ESC male, ESC female, PGC male, and PGC female. (B) Boxplot showing the distribution of gene expression levels across ESC male, ESC female, PGC male, and PGC female. Volcano plot of DEGs between ESC male_ vs_ PGC male (C) and ESC female _vs _PGC female (D) comparisons. (E) GO classification of DEGs related to metabolic processes. (F) Proportion of metabolic process-related DEGs in male and female samples. GO enrichment analysis of DEGs from ESC male _ vs_ PGC male (G) and ESC female_ vs_ PGC female (H) samples. ESC, embryonic stem cell; PGC, primordial germ cell; DEG, differentially expressed gene; GO, Gene Ontology.

Illumina NovaSeq 6000 sequencing generated 90.12 Gb of raw data and 585,752,966 raw reads from ESC and PGC samples. FastQC analysis revealed that the Q30 scores for all samples ranged from 92.87% to 93.78%, indicating high sequencing data quality. After trimming adapter sequences and low-quality reads, a total of 517,031,606 clean reads were obtained, with an average of 88.27% of the clean reads successfully mapped to the reference genome, identifying 14,405 genes. The gene expression levels obtained from transcriptome sequencing are provided in Supplement 2. QC of the sequencing results was performed using PCA (Figure 4A), showing that the three biological replicates in each group were tightly clustered. The first two principal components explained 90.09% of the total variance (PC1 = 58.67%, PC2 = 31.42%), indicating high reproducibility of the transcriptomic data. Additionally, box plots of gene expression across multiple samples exhibited high consistency (Figure 4B), confirming uniform gene expression distribution, good sequencing quality, and proper normalization, thereby enabling further analysis.

Volcano plot analysis revealed a similar number of dferentially expressed genes (DEGs) in both comparisons (ESC male vs. PGC male and ESC female vs. PGC female). A total of 4,328 DEGs were identified in males, while 4,122 DEGs were identified in females (Figures 4C, 4D). GO functional annotation was performed to categorize the DEGs in ESC and PGC samples, showing similar expression patterns between male and female samples. All DEGs were successfully annotated into GO functional categories: “Cellular Component,” “Molecular Function,” and “Biological Process.” The DEGs were distributed into five main categories: “Cellular Processes,” “Environmental Information Processing,” “Genetic Information Processing,” “Human Diseases,” “Metabolism,” and “Organismal Systems.” The majority of DEGs were classified under the metabolism category, with 7,404 genes related to metabolic processes (Figure 4E). Metabolism-related DEGs accounted for 49.51% in males and 49.25% in females, respectively, among the total DEGs (Figure 4F). This similar proportion indicates a high degree of consistency in metabolic pathway changes during PGC formation, further validating the central role of metabolic regulation in PGC development. Within the metabolism category, the top three significantly enriched pathways were “Amino Acid Metabolism,” “Lipid Metabolism,” and “Carbohydrate Metabolism” (Figures 4G, 4H).

### Integrated analysis of dfferentially expressed genes and differentially expressed metabolites

**Figure 5:**
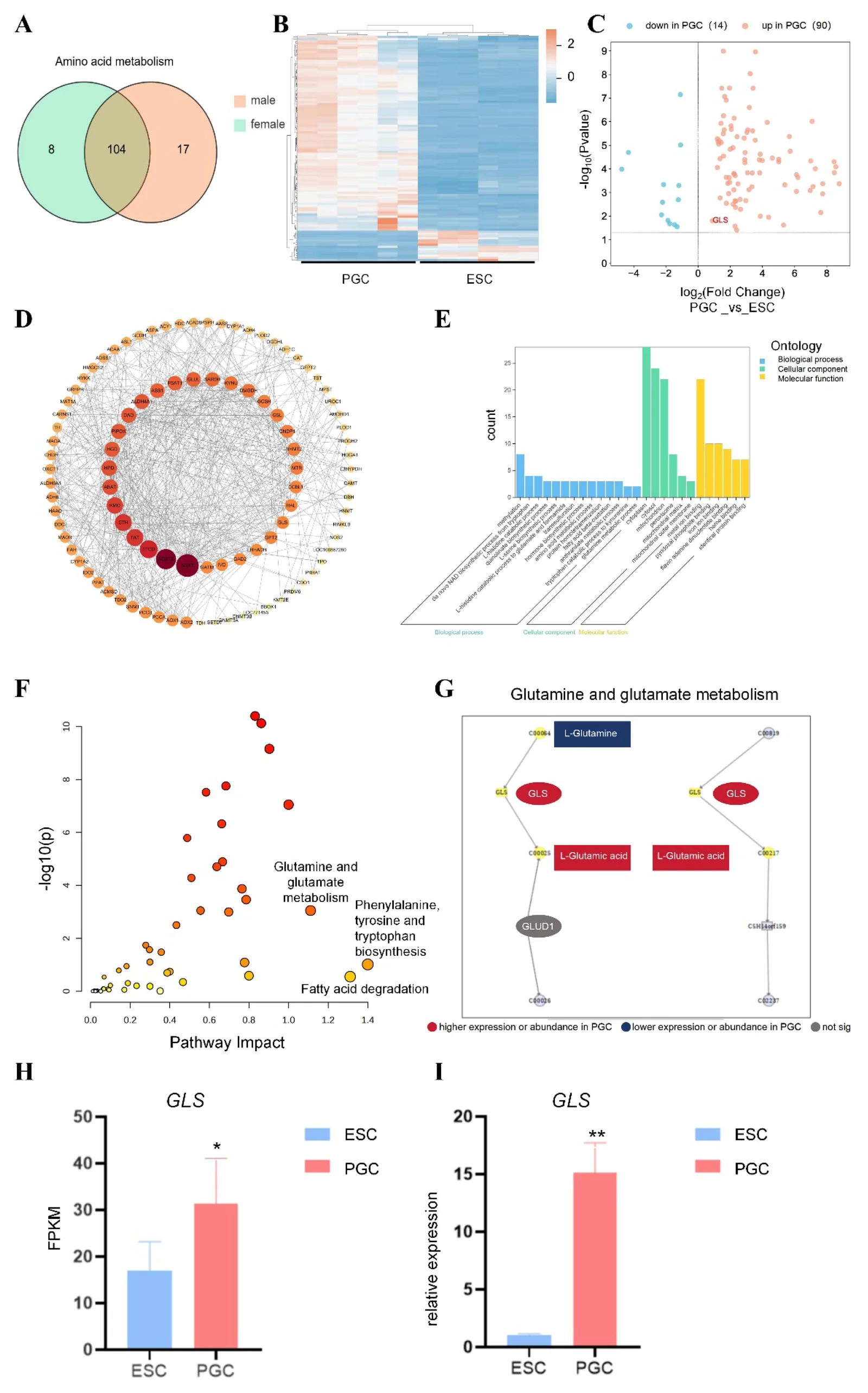
Comprehensive analysis of amino acid metabolism-related DEGs and their role in PGC formation. (A) Venn diagram showing the overlap of DEGs involved in amino acid metabolism. (B) Clustering heatmap showing the expression patterns of amino acid metabolism-related DEGs during PGC formation. (C) Volcano plot of amino acid metabolism-related DEGs during PGC formation. (D) PPI network constructed based on STRING database for the DEGs involved in amino acid metabolism. (E) GO enrichment analysis of amino acid metabolism-related DEGs in the PPI network. (F) Pathway impact analysis of amino acid metabolism-related DEGs and DEMs. (G) KEGG pathway mapping showing the overlap between DEGs and DEMs in the top three enriched metabolic pathways. Yellow labels indicate molecules detected by multiomics sequencing; red indicates molecules with relatively higher expression or abundance in PGC; blue indicates molecules with relatively lower expression or abundance in PGC; and gray indicates non-significant changes. (H) Expression of GLS, a gene involved in glutamine metabolism, during PGC formation (data are shown as mean±SEM, differential expression was identified by DESeq2 (* p<0.05). (I) The expression of GLS was detected by qRT-PCR (data are shown as mean±SEM, n = 3 independent experiments, *** p<0.001 Student’s t-test). Asterisks indicate significance from the global test (* p<0.05, ** p<0.01). PGC, primordial germ cell; ESC, embryonic stem cell; DEG, differentially expressed gene; PPI, protein-protein interaction; GO, Gene Ontology; DEMs, differentially expressed metabolites; KEGG, Kyoto Encyclopedia of Genes and Genomes; SEM, standard error of the mean; qRT-PCR, quantitative real-time polymerase chain reaction.

Metabolomics analysis indicated that amino acid metabolism undergoes significant changes during the differentiation of ESC into PGC, suggesting that genes related to amino acid metabolism also exhibit substantial alterations in expression. To exclude the influence of sex-related factors on differential gene expression, a Venn analysis was performed, identifying 104 common DEGs involved in amino acid metabolism (Figure 5A). Further cluster heatmap analysis and volcano plot revealed that most of the genes related to amino acid metabolism were expressed at low levels in ESC and at high levels in PGC, which was consistent with the results of metabolomics sequencing (Figures 5B, 5C). This finding suggests that enhanced amino acid metabolic activity may be a key feature of PGC development.

To explore potential interactions among differentially expressed proteins, a PPI network was constructed using the STRING database, followed by GO functional annotation. The results revealed that the PPI network consisted of 97 nodes and 590 edges, with an average degree of 12.165 (Figure 5D), indicating extensive interactions among the differentially expressed proteins, forming a tightly connected functional network. These genes were enriched in pathways related to tryptophan metabolism, serine metabolism, histidine metabolism, and glutamine metabolism (Figure 5E).

To investigate the correlation between amino acid metabolism-related DEGs and DEMs during PGC formation, an integrated analysis of the sequencing data was conducted. The top three pathways based on pathway impact scores were phenylalanine, tyrosine, and tryptophan biosynthesis, fatty acid degradation, and glutamine and glutamate metabolism (Figure 5F). Moreover, DEGs and DEMs from these three pathways were mapped to the KEGG database to identify their shared pathways, with the results showing that most DEMs were significantly positively or negatively correlated with DEGs (Figure 5G). Notably, *GLS*, encoding the key enzyme for glutamine degradation, was expressed at a significantly higher level in PGC than in ESC during PGC formation according to the transcriptomic data (Figure 5H; * p< 0.05). Consistent with the RNA sequencing (RNA-seq) results, qRT-PCR analysis further confirmed that *GLS* expression was significantly higher in PGC than in ESC (Figure 5I; *** p<0.001). Based on these findings, we conclude that amino acid metabolism, particularly glutamine catabolism, plays a crucial role in PGC formation.

### Glutamine promotes primordial germ cell-like cell specification

**Figure 6:**
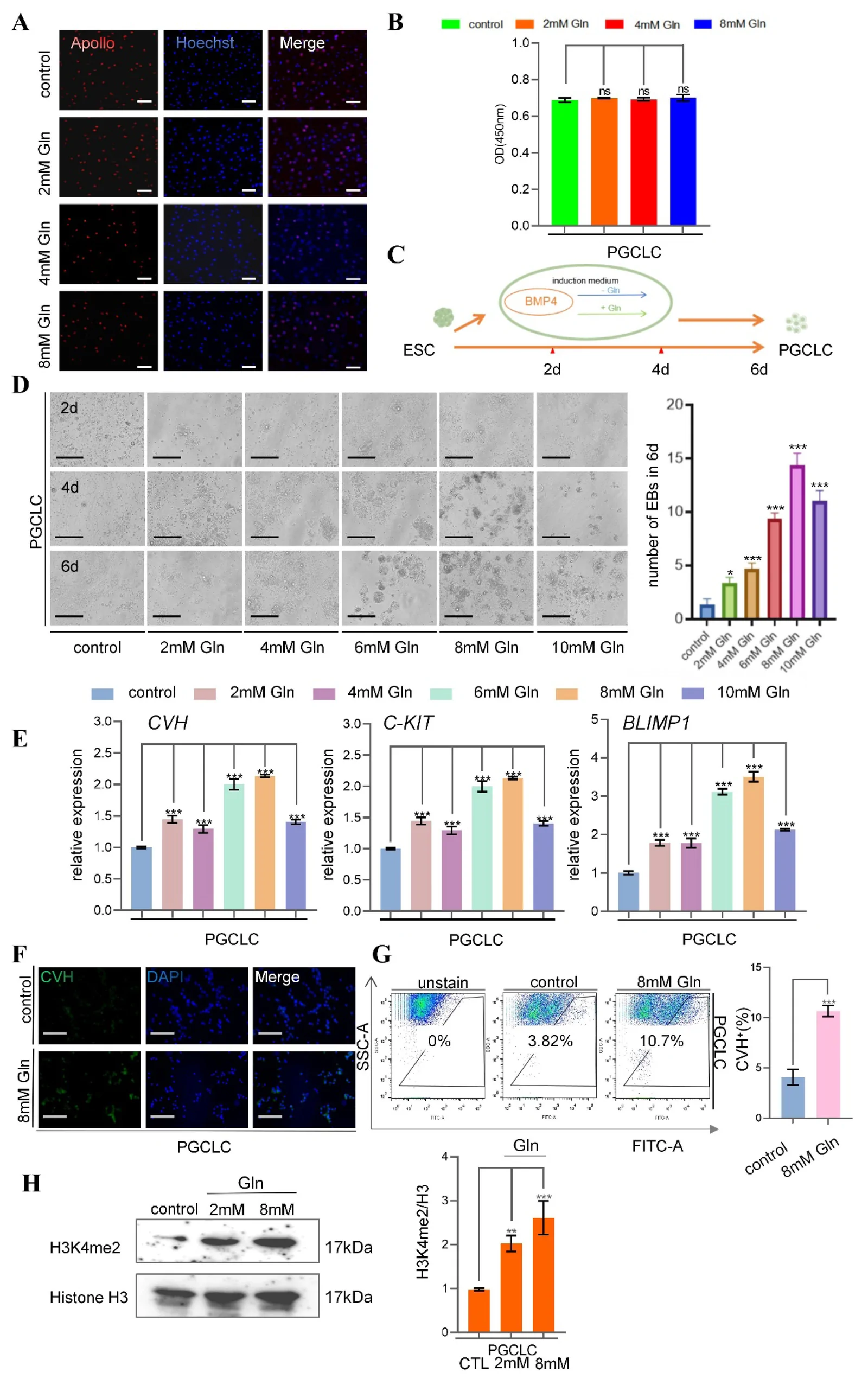
Glutamine promotes differentiation of ESC into PGC. (A) After adding different concentrations of Glutamine *in vitro*, EDU detects the effect of DF-1 cell. Scale bar: 100 μm (n = 3 independent experiments). (B) The effects of different concentrations of on the proliferation of DF-1 cell were detected by CCK-8 assay (data are shown as mean±SEM, n = 3 independent experiments, ns p>0.05 one-way analysis of variance). (C) The schematic diagram of PGC induced from chicken ESC *in vitro*. (D) Left: Morphological observation of the number of embryoid bodies in the PGC induction model after adding 8 mM Gln *in vitro*; BMP4 induction in glutamine-deficient medium was used as a control, Scale bar: 60 μm (n = 3 independent experiments); Right: Quantification of EB numbers in the BMP4-induced differentiation model across glutamine concentration gradients (data are shown as mean±SEM, n = 3 independent experiments, * p<0.1, *** p<0.001 one-way analysis of variance). (E) After adding 8 mM Gln *in vitro*, the expression of PGC marker genes CVH, C-KIT and BLIMP1 in PGCLC was detected by qRT-PCR (data are shown as mean±SEM, n = 3 independent experiments, *** p<0.001 one-way analysis of variance). (F) 6 d after induction, the efficiency of PGCLC formation by adding 8 mM Gln *in vitro* was detected by IF using CVH+ labeled protein. Scale bar: 200 μm (n = 3 independent experiments). (G) At 6 d after induction, the efficiency of PGCLC formation after the addition of 8 mM Gln was evaluated by flow cytometry using CVH+ marker proteins (data are shown as mean±SEM, n = 3 independent experiments, *** p<0.001 Student’s t-test). (H) Levels of H3K4me2 in PGCLC after 6 d of induction with different concentrations of Gln *in vitro* (data are shown as mean±SEM, n = 3 independent experiments, ** p<0.01, *** p<0.001 one-way analysis of variance). PGCLC, primordial germ cell-like cell; ESC, embryonic stem cell; EBs, embryoid bodies; PGC, primordial germ cell; EdU, 5-ethynyl-2′-deoxyuridine; CCK-8, cell counting kit-8; SEM, standard error of the mean; qRT-PCR, quantitative real-time polymerase chain reaction; IF, immunofluorescence.

To investigate the role of glutamine in PGC formation, this study utilized a BMP4-induced model previously established in our laboratory. The goal was to induce ESC differentiation into PGCLC under glutamine-deprived conditions and assess the efficiency of PGCLC specification. Initially, to exclude the toxic effects of high concentrations of glutamine on the cells, DF-1 cells were chosen as the model system. Different concentrations of glutamine were added during cell culture to evaluate cell proliferation and apoptosis, and the optimal glutamine concentration was selected. EdU and CCK-8 were performed to identify glutamine concentrations that did not affect ESC proliferation. The results showed no significant differences between groups, confirming normal cell proliferation (Figures 6A, 6B; ns p>0.05). EdU staining revealed no noticeable differences in green fluorescence between groups (Figure 6A). Consistent with the EdU staining results, CCK-8 assays indicated no significant differences between groups, further confirming normal cell proliferation (Figure 6B; ns p>0.05). These results suggest that glutamine, as a NEAA, has low cytotoxicity and does not adversely affect cell proliferation or apoptosis.

In subsequent phases, a BMP4 induction system was employed, using a 2 mM gradient of glutamine to induce ESC differentiation into PGCLC (Figure 6C). The growth state of the cells in different groups was compared by observing cell morphology. The results showed that, compared to the control group, embryoid bodies (EBs) began to form on 4 d in the control group, while in the glutamine-supplemented group, EBs began to form on 2 d. The number of EBs significantly increased by 4 d and peaked on 6 d. Random statistical analysis of the number of EBs in three fields of view (Figure 6D) showed a dose-dependent increase in EBs numbers, with the maximum number of EBs observed at a glutamine concentration of 8 mM (Figure 6D, * p<0.1, *** p<0.001).

Quantitative qRT-PCR was used to assess the expression levels of PGC marker genes *CVH*, *C-KIT*, and *BLIMP1*. The results indicated that, compared to the control group, the expression of *CVH*, *C-KIT*, and *BLIMP1* was significantly upregulated after glutamine supplementation (Figure 6E; *** p<0.001), with the highest relative expression observed at 8 mM, suggesting that exogenous glutamine supplementation enhances the efficiency of PGCLC specification. Furthermore, indirect immunofluorescence (IF) and FC analyses confirmed that the glutamine-supplemented group had an increased proportion of CVH-positive cells (Figures 6F, 6G; *** p< 0.001). Given the intimate relationship between cellular metabolism and epigenetic regulation during cell fate determination, we further examined the level of H3K4me2, a histone modification previously reported to facilitate PGCLC specification [10]. Western blot analysis showed that glutamine supplementation was accompanied by a significant increase in H3K4me2 levels during PGCLC induction (Figure 6H; ** p<0.01; *** p<0.001). This finding suggests that, in addition to supporting cellular metabolism, glutamine supplementation may be associated with epigenetic changes that accompany germ-cell lineage commitment. Taken together, these results demonstrate that glutamine promotes PGCLC specification and is accompanied by increased H3K4me2 levels, highlighting a potential link between glutamine metabolism and epigenetic regulation during germ-cell development.

### Glutamine may influence oxidative phosphorylation potentially via its metabolite α-ketoglutarate during primordial germ cell-like cell specification

**Figure 7:**
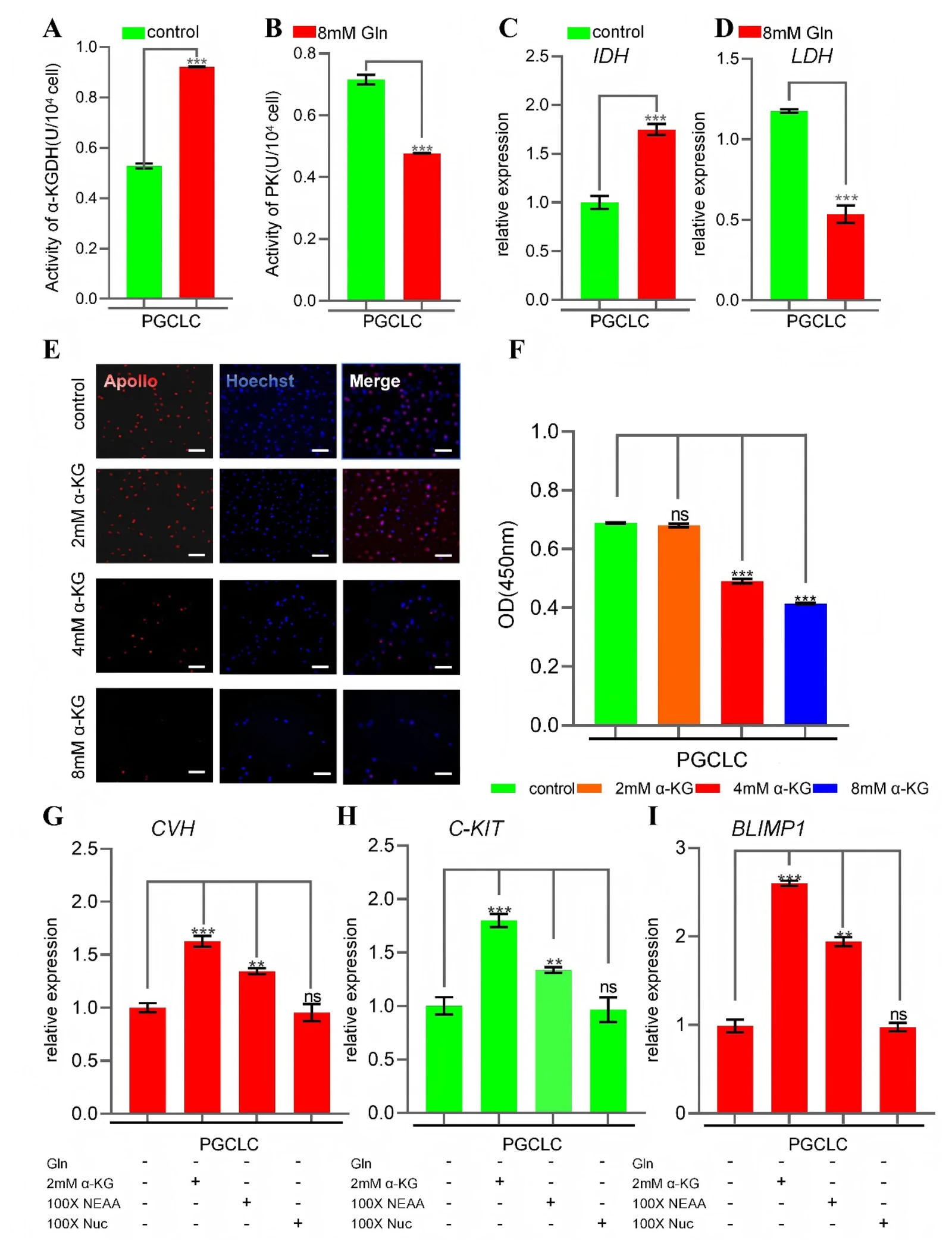
Influence of glutamine on oxidative phosphorylation and epigenetic modifications in PGCLC. (A) Enzymatic activity of α-KGDH in cells after the addition of 8 mM Gln *in vitro*. BMP4 induction in glutamine-deficient medium serves as the control (data are shown as mean±SEM, n = 3 independent experiments, *** p<0.001 Student’s t-test.). (B) Enzymatic activity of pyruvate kinase (PK) in cells after the addition of 8 mM Gln *in vitro* (data are shown as mean±SEM, n = 3 independent experiments, *** p<0.001 Student’s t-test). Expression of oxidative phosphorylation-related gene IDH (C) and glycolysis-related gene LDH (D) in PGCLC after the addition of 8 mM Gln *in vitro*, detected by qRT-PCR (data are shown as mean±SEM, n = 3 independent experiments, *** p<0.001 Student’s t-test). (E) After adding different concentrations of α-KG *in vitro*, EDU detects the effect of DF-1 cell. Scale bar: 100 μm (n = 3 independent experiments). (F) The effects of different concentrations of on the proliferation of DF-1 cell were detected by CCK-8 assay (data are shown as mean±SEM, n = 3 independent experiments, ns p>0.05, *** p<0.001 one-way analysis of variance). (G–I) Expression of primordial reproductive marker genes CVH, C-KIT, and BLIMP1 in PGCLC after the addition of 2 mM DM-α-KG (α-KG), 100X NEAA, and 100X NuC, respectively, in the absence of Gln *in vitro*, detected by qRT-PCR (data are shown as mean±SEM, n = 3 independent experiments, ns p>0.05, ** p<0.01, *** p<0.001 one-way analysis of variance). α-KGDH, α-ketoglutarate dehydrogenase; PGCLC, primordial germ cell-like cell; OD, optical density; SEM, standard error of the mean; qRT-PCR, quantitative real-time polymerase chain reaction; EdU, 5-ethynyl-2′-deoxyuridine; CCK-8, cell counting kit-8; DM-α-KG, dimethyl α-ketoglutarate; NEAA, non-essential amino acid.

During the differentiation of ESC into PGC, there is a transition in cellular energy metabolism from glycolysis to OXPHOS. Although glutamine is recognized as a key precursor in the TCA cycle for OXPHOS, it remains unclear whether glutamine influences the glycolysis–OXPHOS balance during PGC formation. To address this, glutamine was added to the BMP4 induction medium, and the enzyme activities of the key rate-limiting enzyme α-KGDH in OXPHOS and PK, a glycolysis-related enzyme, were measured by ELISA. The results showed that, compared to the control group, glutamine supplementation significantly increased α-KGDH activity while decreasing PK activity (Figures 7A, 7B; *** p<0.001). Furthermore, qRT-PCR analysis revealed that, after glutamine supplementation, the relative expression of the OXPHOS-related gene *IDH* was significantly increased, while the expression of the glycolysis-related gene *LDH* was significantly reduced (Figures 7C, 7D; *** p<0.001). These findings confirm that exogenous glutamine contributes to PGCLC specification by enhancing OXPHOS levels and reducing glycolytic activity.

It is noteworthy that glutamine can be converted into nucleotides, NEAAs, and α-KG during energy metabolism [26]. α-KG, as a key intermediate in the TCA cycle, plays a role in regulating the cellular glycolysis–OXPHOS balance. Therefore, we hypothesized that glutamine is metabolized into α-KG through the TCA cycle, which in turn regulates the OXPHOS levels in PGC, promoting their lineage-specific differentiation. To validate the role of α-KG in rescuing PGCLC specification in glutamine-deprived induction medium, various downstream derivatives were introduced. Dimethyl α-ketoglutarate (DM-α-KG), a low-toxicity permeable compound, was tested at a concentration of 2 mM (Figures 7E, 7F; ns p>0.05; *** p<0.001). qRT-PCR results demonstrated that α-KG effectively replaced glutamine and significantly upregulated the relative expression of PGC marker genes *CVH*, *C-KIT*, and *BLIMP1*, promoting lineage-specific PGC differentiation. In contrast, attempts to rescue PGCLC specification using nucleotides and NEAAs were unsuccessful (Figures 7G–7I; ns p>0.05; ** p<0.01; *** p<0.001).

**Figure 8:**
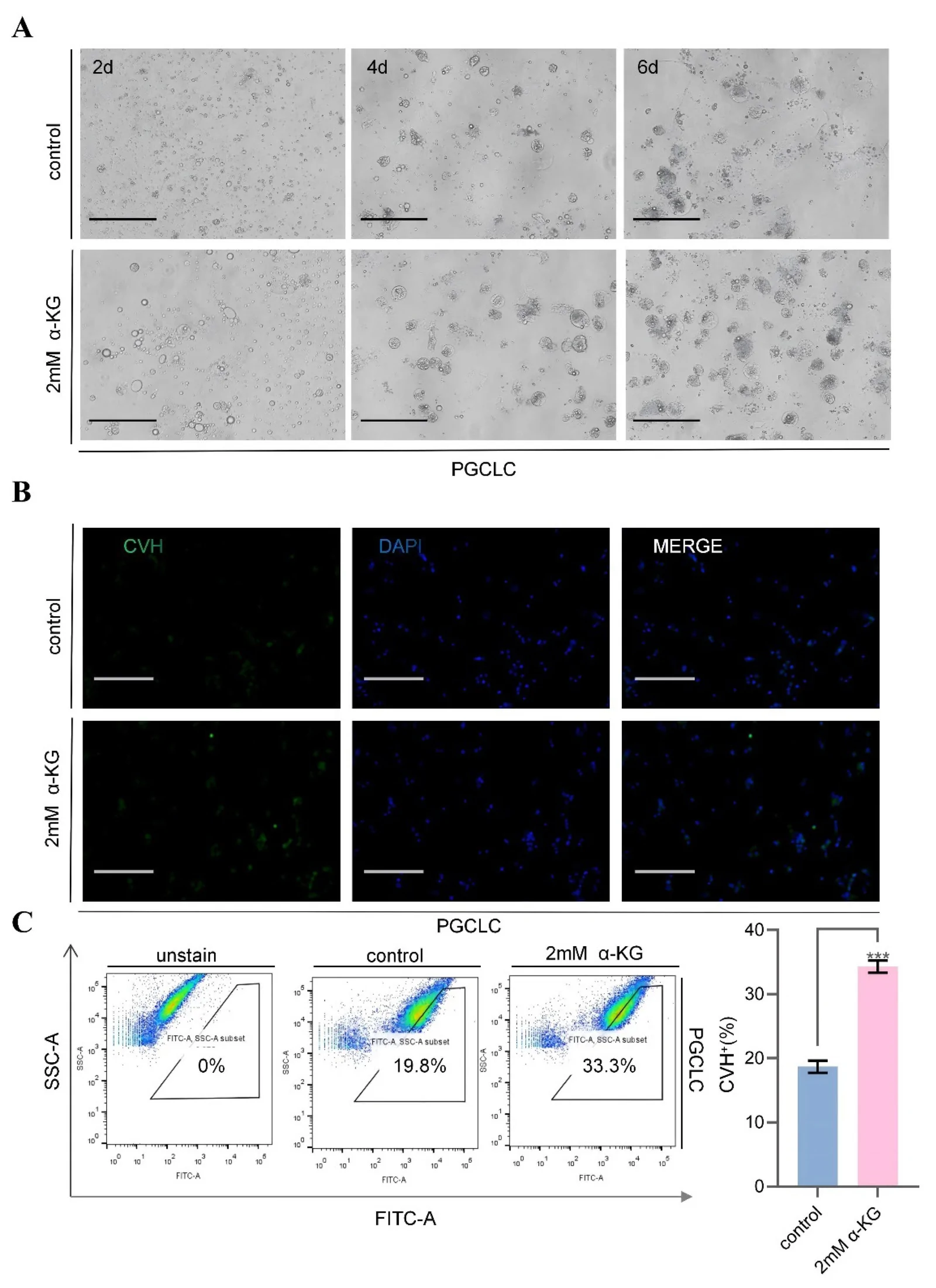
α-KG, a downstream derivative of glutamine TCA, promotes the fate decision of PGC. (A) Morphological observation of the number of embryoid bodies in the PGCLC induction model after adding 2 mM α-KG *in vitro*; BMP4 induction in a glutamine-deficient medium was used as a control. Scale bar: 60 μm (n = 3 independent experiments). (B) 6 d after induction, the efficiency of PGCLC formation by adding 2 mM α-KG *in vitro* was detected by IF using CVH+ labeled protein. Scale bar: 200 μm (n = 3 independent experiments). (C) At 6 d after induction, the efficiency of PGCLC formation after the addition of 2 mM α-KG was evaluated by FC using CVH+ marker proteins (data are shown as mean±SEM, n = 3 independent experiments, *** p<0.001 Student’s t-test). α-KG, α-ketoglutarate; PGCLC, primordial germ cell-like cell; TCA, tricarboxylic acid; PGC, primordial germ cell; IF, immunofluorescence; FC, flow cytometry; SEM, standard error of the mean.

To further confirm that α-KG can rescue the reduced PGCLC specification efficiency caused by glutamine depletion, this study added α-KG, a downstream derivative of glutamine, to glutamine-deficient culture medium and used BMP4 to induce ESC differentiation into PGCLC. Cell morphology was observed to compare the growth status of the different groups in the culture medium. The results showed that, after the addition of α-KG, EBs began to form on 2 d, whereas in the control group, EBs only started forming on 4 d. Furthermore, compared to the control group, the number of EBs in the α-KG supplementation group was significantly increased on 2, 4, and 6 d (Figure 8A). Additionally, indirect IF and FC were used to assess PGCLC specification efficiency, and the results showed that the number of CVH-positive cells in the α-KG supplementation group was significantly higher than that in the control group (Figures 8B, 8C; *** p<0.001), indicating that α-KG can actively regulate PGCLC specification in the absence of exogenous glutamine. These results demonstrate that glutamine promotes PGCLC specification, potentially through its downstream metabolite α-KG.

## DISCUSSION

PGC plays a crucial role in reproductive health, as their development is closely associated with the maintenance of reproductive capacity and the transmission of genetic information [27]. In recent years, increasing evidence has revealed significant changes in metabolites during germ cell development. Our study identified a total of 194 differentially DEMs common to both male and female samples, including amino acids, peptides and their analogs, carbohydrates, glycerophosphocholine, glycerophosphoethanolamine, and fatty acids and their conjugates. These DEMs were significantly enriched in the FoxO signaling pathway and in glutamine and glutamate metabolism. We hypothesize that oxidative stress, metabolic homeostasis, and glutamine metabolism influence PGC formation. PCA and HCA revealed distinct metabolic profiles between ESC and PGC, indicating that metabolite accumulation patterns may change during PGC formation. Specifically, amino acids, peptides, and analogs were more abundant in PGC, while carbohydrates and their conjugates were more abundant in ESC. This suggests that amino acid metabolism is more active in PGC, while ESC may have higher energy metabolic demands and enhanced glycolytic activity, a finding consistent with previous studies [28,29].

Interestingly, functional annotation of these DEGs revealed significant enrichment in “Amino acid metabolism,” “Lipid metabolism,” and “Carbohydrate metabolism.” This further supports the notion that amino acid and glucose metabolism activities change during PGC development [19,30]. We also observed a significant upregulation in the expression of genes associated with amino acid metabolism, which may be closely related to the protein synthesis requirements during PGC formation. Amino acids not only serve as building blocks for proteins but also participate in regulating cellular functions through various metabolic pathways [31,32]. Changes in lipid and carbohydrate metabolism may be involved in important biological processes such as membrane synthesis, signal transduction, and energy metabolism, all of which are crucial for cell proliferation, differentiation, and long-term stability [33,34].

Comprehensive analysis of DEGs and DEMs indicated that tryptophan metabolism, serine metabolism, histidine metabolism, and glutamine metabolism underwent significant changes during ESC differentiation into PGC. Previous studies have demonstrated that these amino acids are crucial for maintaining the health of reproductive cells and normal reproductive function. For example, tryptophan regulates oocyte function and female fertility by contributing to the de novo synthesis of NAD [34,35]; serine metabolism plays a central role in one-carbon metabolism, supporting DNA and RNA synthesis, which is essential for the rapid proliferation of reproductive cells [36]. Moreover, the antioxidant capabilities of histidine and glutamine are particularly important in reproductive cells [37,38], and our findings further support this notion. Notably, while glutamine is lowly expressed in PGC, its downstream metabolite, glutamate, is highly expressed, and the gene GLS, involved in glutamine catabolism, is significantly upregulated during PGC formation, suggesting that glutamine metabolism plays a role in PGC formation.

Although glutamine is classified as a NEAA, recent studies have clarified its critical role in the lineage specificity of germ cells [12,39,40]. Our study further confirms the importance of glutamine metabolism in the determination of germ cell lineages. Under the unique induction conditions for PGC and the context of cell fate determination, we observed that glutamine acts similarly to an essential amino acid, particularly in promoting reproductive lineage differentiation. Our results are consistent with the current understanding that OXPHOS is the main energy-generating pathway in PGC [19]. Previous research has primarily focused on the effects of α-KG, a downstream metabolite of glutamine, on the self-renewal of pluripotent stem cell. For instance, α-KG has been shown to enhance self-renewal and inhibit differentiation by promoting histone and DNA demethylation [12]. However, studies involving the addition of α-KG to human pluripotent stem cell (hPSCs) under standard culture conditions (both in the naïve and more developmentally mature pluripotent states) have revealed similar mechanisms, though with opposite results [41]. Notably, our study found that under BMP4 induction, α-KG promoted the differentiation of ESC into PGC, further supporting the dual effects of α-KG on both self-renewal and differentiation under different conditions. It is worth emphasizing that the findings of this study support a specific role for α-KG in glutamine-mediated PGCLC specification. Under glutamine-depleted conditions, only α-KG was able to partially restore PGCLC specification, whereas other downstream metabolites of glutamine, including nucleotides and non-essential amino acids, failed to produce a similar effect. This suggests that the process does not rely solely on the replenishment of biosynthetic substrates or the restoration of energy metabolism, but rather may involve α-KG-mediated metabolic-epigenetic coupling regulation.

It is worth noting that α-KG is widely recognized as an essential cofactor for α-KG-dependent dioxygenases, including JmjC domain-containing histone demethylases, which are generally associated with the removal of methyl groups from H3K4 [42]. However, glutamine supplementation in the present study was accompanied by an increase in H3K4me2 levels. This observation suggests a potential association between glutamine metabolism and H3K4 methylation status during PGCLC specification. The mechanism underlying this increase in H3K4me2 remains unclear. Previous studies have reported that certain JmjC domain-containing proteins are capable of regulating transitions among different H3K4 methylation states, including the conversion of H3K4me3 to H3K4me2 [43]. Therefore, it is possible that increased α-KG availability may influence the dynamic balance of H3K4 methylation states during differentiation. In addition, H3K4me2 has been reported to be associated with transcriptional competence, lineage priming, and developmental gene activation [10,43,44]. Thus, the elevated H3K4me2 levels observed in this study may reflect chromatin-state changes occurring during ESC-to-PGCLC differentiation. Collectively, these findings suggest that the glutamine-α-KG metabolic axis may contribute to the pre-activation of germline-related genes and the establishment of PGC fate by modulating cellular metabolic status.

It should be noted that the multi-omics analyses in this study were performed on freshly isolated embryonic PGC, whereas the functional and mechanistic studies were primarily conducted using a BMP4-induced PGCLC *in vitro* model. Although this model effectively recapitulates several key features of early germline lineage specification and has been widely used to study the mechanisms of early germ cell fate determination, it cannot fully reproduce the complex microenvironment, cell-cell interactions, and dynamic metabolic regulation present during *in vivo* embryonic development. Therefore, the proposed glutamine-driven regulatory mechanism involving potential changes in α-KG metabolism requires further validation of its applicability under physiological *in vivo* conditions.

## CONCLUSION

This study combined metabolomic and transcriptomic analyses to compare chicken ESC and PGC, revealing notable changes in amino acid metabolism during PGC formation. Our results suggest that glutamine contributes to chicken PGCLC specification, with associated changes in cellular metabolism that may be associated with metabolic pathways such as OXPHOS and potentially its metabolite α-KG. While these findings provide mechanistic insights into the metabolic of germ cell development, further functional validation and *in vivo* studies are needed to confirm their broader applicability. Future research could explore how modulating glutamine availability or related metabolic pathways may optimize PGC culture conditions and improve strategies for poultry germline preservation.

## Figures and Tables

**Table 1:** Composition of differentiation induction medium for control (glutamine-deficient), Gln (glutamine-sufficient), and α-KG (α-KG rescue) conditions

Component	Control	Gln	α-KG
High-glucose DMEM, no glutamine	+	+	+
10% FBS	+	+	+
2.5% chicken serum	+	+	+
100 U/mL penicillin	+	+	+
100 U/mL streptolysin	+	+	+
0.1 mmol/Lβ-mercaptoethanol	+	+	+
40 ng/mL BMP4 protein	+	+	+
2 mmol/L glutamine	-	+	-
2 mmol/L DM-α-KG	-	-	+

α-KG, α-ketoglutarate; DMEM, Dulbecco’s modified Eagle medium; FBS, fetal bovine serum; DM-α-KG, dimethyl α-ketoglutarate.

**Table 2:** Real-time quantitative PCR primer information table

Gene	Primer sequence
GLS	F: 5′-GTCGCCTCCTTTGGACAAGA-3′ R: 5′-CACTGACTTGACCCGCTGAT-3′
CVH	F: 5′-AGGAGGACTGGGACACG-3′ R: 5′-GCCTCTTGATGCTACCG-3′
C-KIT	F: 5′-GCGAACTTCACCTTACCCGATTA-3′ R: TGTCATTGCCGAGCATATCCA -3′
BLIMP1	F: 5′-ATGAAGGCTGCTACACGG-3′ R: 5′-GCAGTTTGATGCGTATTTG-3′
IDH	F: 5′-AGGCCTGCCTAATGTCACAC-3′ R: 5′-CCAGCTTCCCCTTCAGGTTT-3′
LDH	F: 5′-TGGGCATCCATCCTCTGA-3′ R: 5′-CCTGCTTGTGAACCTCCT-3′
β-ACTIN	F: 5′-CAGCCATCTTTCTTGGGTAT-3′ R: 5′-CTGTGATCTCCTTCTGCATCC-3′

PCR, polymerase chain reaction.

## SUPPLEMENTARY MATERIAL

**Supplement 1.** Metabolomics sequencing data.

**Supplement 2.** Transcriptomics sequencing data.
